# Tumor-related neurocognitive dysfunction in patients with diffuse glioma: a systematic review of neurocognitive functioning prior to anti-tumor treatment

**DOI:** 10.1007/s11060-017-2503-z

**Published:** 2017-05-31

**Authors:** Emma van Kessel, Anniek E. Baumfalk, Martine J. E. van Zandvoort, Pierre A. Robe, Tom J. Snijders

**Affiliations:** 1 Department of Neurology & Neurosurgery, University Medical Center Utrecht/Brain Center Rudolf Magnus, G03.232, PO Box 85500, 3508 XC Utrecht, The Netherlands; 2 Helmhotz Institute, Utrecht University, Room 1715, Heidelberglaan 1, 3584 CS Utrecht, The Netherlands

**Keywords:** Neurocognitive functioning, Cognition, Glioma, Neuropsychology, Brain tumor

## Abstract

Deficits in neurocognitive functioning (NCF) frequently occur in glioma patients. Both treatment and the tumor itself contribute to these deficits. Data about the role of the tumor are scarce, because NCF has mostly been studied postoperatively. We aimed to summarize data on pre-treatment NCF in glioma patients and to determine the overall and domain-specific prevalence of neurocognitive dysfunction. We searched PubMed and Embase according to PRISMA-P protocol for studies that evaluated pre-treatment NCF in glioma patients (1995-November 2016) and extracted information about NCF. We performed analysis of data for two main outcome measures; mean cognitive functioning of the study sample (at group level) and the percentage of impaired patients (at individual level). We included 23 studies. Most studies were small observational prospective cohort studies. In 11 (47.5%) studies, patient selection was based on tumor location. NCF was analyzed at the group level in 14 studies, of which 13 (92.9%) found decreased NCF at group level, compared to normative data or matched controls. The proportion of individuals with decreased NCF was reported in 15 studies. NCF was impaired (in any domain) in 62.6% of the individuals (median; interquartile range 31.0–79.0). Cognitive impairments were more common in patients with high-grade glioma than with low-grade glioma (OR 2.50; 95% CI 1.71–3.66). Cognitive impairment occurs in the majority of treatment-naive glioma patients, suggesting that neurocognitive dysfunction is related to the tumor. However, the literature about pre-treatment NCF in glioma patients is characterized by small-scale studies and strong heterogeneity in patient selection, resulting in high risk of bias.

**Electronic supplementary material:**

The online version of this article (doi:10.1007/s11060-017-2503-z) contains supplementary material, which is available to authorized users.

## Introduction

Diffuse gliomas are progressive brain tumors that are almost invariably fatal, despite recent advances in treatment. Patients with diffuse glioma (WHO grade II–IV) often have deficits in neurocognitive functioning (NCF). Frequently reported problems concern word-finding, short-term memory and carrying out complex tasks [1–4]. Impairments in NCF negatively influence health-related quality of life (HRQOL) of patients and their partners [5–7]. For this reason, NCF is an important outcome measure of treatment, especially in patients with low-grade glioma (LGG, WHO grade II), who show relatively long periods of disease-free survival compared to patients with high-grade glioma (HGG, WHO grade III-IV).

The neurocognitive deficits in glioma patients may be found in one or more cognitive domains, such as executive functioning, language and memory. The neurocognitive deficits can be subtle and focal neurological deficits (leg or arm paresis, visual disturbances etc.) can overshadow these neurocognitive problems [5–8]. Therefore, to detect neurocognitive deficits in glioma patients, neuropsychological evaluation with sensitive and wide-ranging tests is required. Standard screening tests aimed at cognitive decay, such as the Folstein Mini Mental State Examination (MMSE) and the Montreal Cognitive Assessment (MoCA), cannot always provide relevant information, because they lack sensitivity and domain-specific information.

Factors affecting NCF can be related to the patient (e.g. age, level of education), the tumor (e.g. tumor grade, tumor location, biologic characteristics of the tumor) and the treatment [1, 9, 10]. The tumor can give rise to neurocognitive deficits through many mechanisms, such as local mechanical effects of the tumor mass and hereby ischemic changes, but also cell death by tumor-released excitotoxins and disturbances in synaptic transmission [11, 12]. These different mechanisms give rise to direct neuronal damage in the region of the tumor, as well as more widespread disturbances of functional brain networks [13]. Because NCF in glioma patients has primarily been studied postoperatively, data about the role of the tumor itself in neurocognitive outcome are scarce [3, 8, 14, 15]. After surgery, the effects of the tumor, surgery and medical therapies on NCF are difficult to distinguish.

Since NCF is an important outcome measure of treatment and the information about the occurrence of neurocognitive deficits in glioma patients before treatment is limited, we performed a systematic literature search to identify studies that evaluated NCF in glioma patients before surgery, chemotherapy or radiotherapy [16]. The aim of this systematic review is to give an overview of data about the occurrence of neurocognitive problems in glioma patients before anti-tumor treatment and about the cognitive domains in which these deficits occur, and to identify gaps of knowledge that may be the subject of future research.

## Methods

We used the PRISMA-P (Preferred Reporting Items for Systematic review and Meta-Analysis Protocols) checklist to ensure good quality of the review (see appendix 1 in supplementary material for the PRISMA-P checklist). Because data from different articles was very heterogeneous and sometimes relevant data was missing, we had to make some important methodological choices. We summarized these choices in box 1 in supplementary material and explain them below.

### Data sources

We performed a search on 24th November 2016 in Pubmed and Embase for articles published between January 1995 and November 2016. The search strategy was developed for PubMed and adapted for Embase. Two search strings were used, one related to gliomas and one related to cognition.

#### Pubmed

(glioma OR “glial cell tumor” OR astrocytoma OR astroglioma OR oligodendroglioma OR oligoastrocytoma OR oligodendroglioma-astrocytoma OR LGG OR HGG OR glioblastoma) AND (cognition OR “cognitive disorders” OR “cognitive function” OR “cognitive functioning” OR “neurocognitive functioning” OR “neurocognitive function” OR “cognitive deficits” OR “cognitive impairment” OR neuropsychologic*).

#### Embase

(glioma:ti,ab OR ‘glial cell tumor’:ti,ab OR astrocytoma:ti,ab OR astroglioma:ti,ab OR oligodendroglioma:ti,ab OR oligoastrocytoma:ti,ab OR oligodendroglioma-astrocytoma:ti,ab OR LGG:ti,ab OR HGG:ti,ab OR glioblastoma:ti,ab) AND (cognition:ti,ab OR ‘cognitive disorders’:ti,ab OR ‘cognitive function’:ti,ab OR ‘cognitive functioning’:ti,ab OR ‘neurocognitive functioning’:ti,ab OR ‘neurocognitive function’:ti,ab OR ‘cognitive deficits’:ti,ab OR ‘cognitive impairment’:ti,ab OR neuropsychologic*:ti,ab).

### Study selection

We included studies that measured NCF prior to surgery, chemotherapy or radiotherapy in adult patients with diffuse infiltrating gliomas of WHO grade II, III and IV. Prior treatment of patients with dexamethasone and/or anticonvulsants was allowed. Only studies that measured NCF with two or more neurocognitive tasks were included. Studies that performed just one test were excluded because these studies generally focus on a specific mean. We excluded case reports, reviews, letters, editorial articles and articles in other languages than English, Dutch, German or French. Studies that only measured NCF with screening tests aimed at cognitive decay/dementia (MMSE or MoCA) were excluded because of the lack of sensitivity and domain-specific information.

Two authors (EvK, AEB) performed selection of the studies. The first selection was based on title and abstract. Relevant articles and papers in which abstracts were missing or which provided insufficient information were retrieved in full text. Study selection was then performed by EvK and AEB and reviewed by TJS. Disagreement between reviewers was resolved through consensus meetings.

### Data extraction

Two authors (EvK, AEB) performed data extraction, with review of data extraction by two authors (TJS, MJEvZ). Data included study items (author, year of publication, study design, selection of study population), patient characteristics (study sample size, sex, age, histology of glioma, WHO grade of glioma, glioma location, treatment) and items of cognitive assessment (mean NCF-scores of the study sample for different neuropsychological tests and the proportion of individual patients with neurocognitive impairment). In case a definition of neurocognitive impairment was not given in the article, it was conservatively defined as a test score of −2 SD or worse, compared to normative data. In appendix 2 in supplementary material we showed which studies described a definition of impairment and in which studies we used conservative definition of impairment (−2SD (<2.5% of the population)). For studies with missing outcome data, the corresponding authors were contacted by e-mail.

To review the available data about neurocognitive functioning in glioma patients for specific domains, we classified the available neuropsychological tests into five cognitive domains: executive & attention, memory, language, visuospatial functioning and (processing) speed (appendix 3 in supplementary material). This classification was supervised by a clinical neuropsychologist with expertise in neuropsychological testing in glioma patients (MJEvZ). To overcome heterogeneity in classification across studies, we made use of predetermined test classification (“domain”) based on state-of-the-art test classification according to international standard and our experience in earlier studies [17].

### Analysis

We summarized the study and patient’s characteristics of the included studies with descriptive summary statistics (Table 1). We performed analysis of data for two main outcome measures:

1. *Group-level* comparison of mean NCF-scores of the patient sample compared to control data or normative data for each neurocognitive test performed (“test level”), for each domain (“domain level”) and for overall neurocognitive dysfunction (“in any domain”)
2. *Individual patient-level* percentage of patients with test performance at the level of impairment; this was calculated for each neurocognitive test performed (“percentage impairment per test”), for each domain (“percentage impairment per domain”) and overall neurocognitive dysfunction (“in any domain”).

**Table 1:** Main characteristics of the included studies

Study characteristic	Value, N (%)
Number of studies	23
Publication year (range)	1997–2016
Study sample size	
Median (IQR)	22 (19–33)
Range	11–233
Study design	
Prospective cohort	16 (69.6%)
Retrospective cohort	5 (21.7%)
Cross-sectional observational	1 (4.3%)
Case-control	1 (4.3%)
Tumor grade	
LGG only	6 (26.1%)
HGG only	2 (8.7%)
LGG and HGG combined	15 (65.2%)
Tumor location	
No selection criteria	12 (52.2%)
Frontal	2 (8.7%)
Temporal	2 (8.7%)
Right hemisphere	1 (4.3%)
Left hemisphere	3 (13.0%)
Eloquent areas	3 (13.0%)
Neuropsychological testing	
No. of tasks used in study	
Median (IQR)	6 (3–10)
Range	2–15
Executive & Attention tested	21 (91.3%)
Memory tested	15 (65.2%)
Language tested	11 (47.8%)
Visuospatial tested	10 (43.5%)
Psychomotor speed tested	15 (65.2%)

*IQR* interquartile range, *LGG* low-grade glioma, *HGG* high-grade glioma

#### Group-level-analysis

In case articles did not directly report on comparison between patient performances and control/normative group, we used one sample T-tests or independent sample T-tests respectively, to statistically test patient performances against norm/control performance per neuropsychological test on the provided data in the study. Then we converted the results from test level to the level of the predetermined domains in the following way: if at group level patients showed a significantly lower score compared to the norm/control data on *any* one of the tests in that particular domain, the respective domain was considered affected.

#### Individual patient-level-analysis

**Fig. 1:**
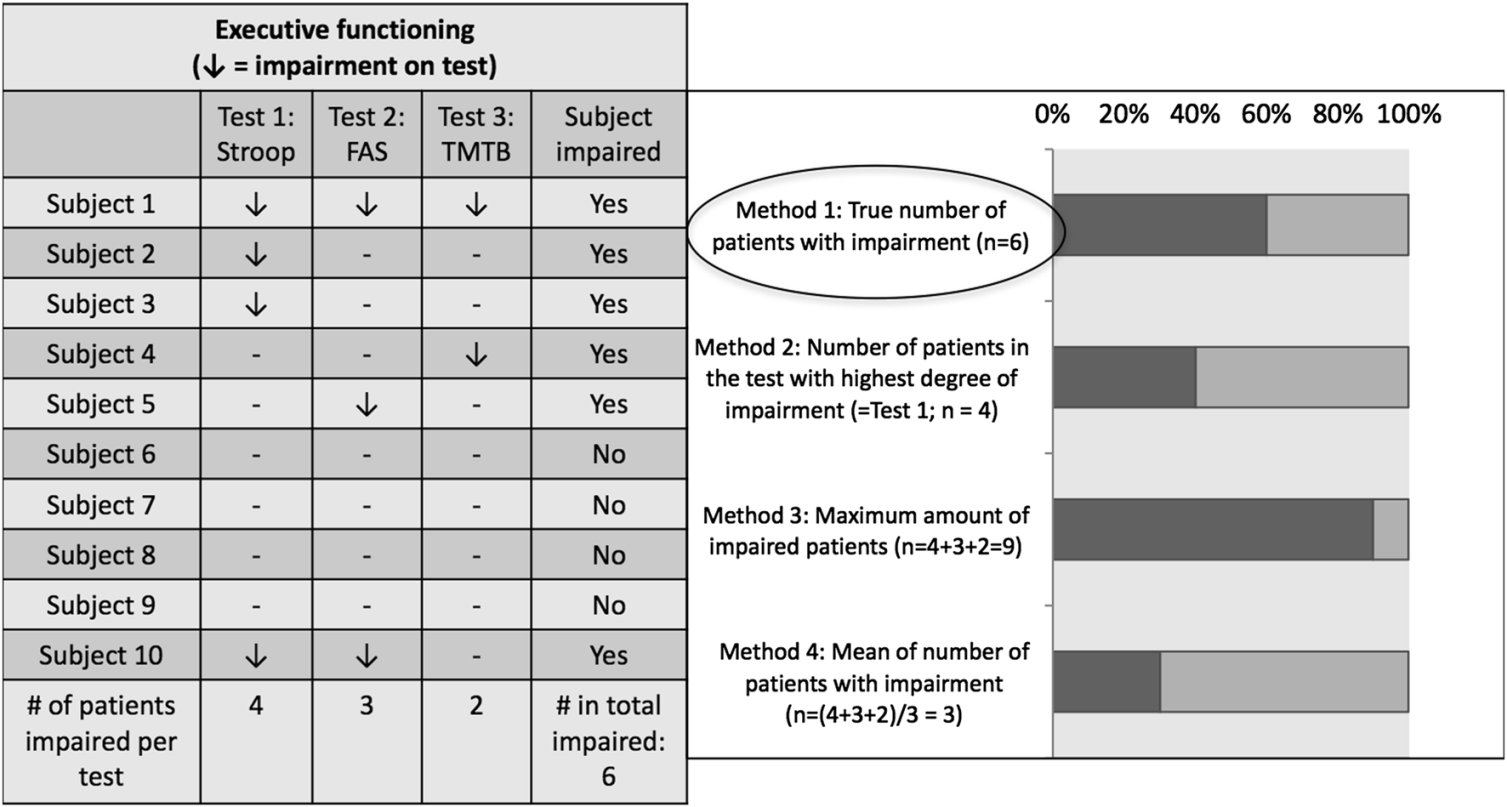
Different methods to calculate the percentage of impaired patients within in a certain cognitive domain (here: executive functioning), based on results from different cognitive tests. Method 1 (true percentage) can only be calculated if individual-level data per test are available. Method 2–4 are based on group results per test: method 2, which is used in this review, will usually give an underestimation of the true proportion, but offers the closest estimation

To determine the percentage impaired patients at the domain level, we counted the number of *individual* patients impaired on the tests within that given domain. A patient was considered impaired for the given domain if he or she performed below threshold on any of the administered tests within the domain. If it had not been stated explicitly whether the given number of impaired patients on a specific test within a domain represented *different individual* patients, and instead only percentages where given per test, we used the results of the test (here named test X) in which most patients were impaired. Hereby the latter number represents the minimum proportion of patients with impairment for the given domain. This most likely is an underestimation of the true proportion, since certain patients may have been unimpaired on test X, but impaired on other tests within that given domain. Figure 1 provides a visual representation of the different methods (and it outcomes) to represent level of impairment. Finally, to summarize the individual patient-level-data across the different studies, we calculated a median with interquartile range (IQR) for each domain.

We also analyzed the relationship between tumor grade (HGG vs. LGG) and frequency of individual-level NCF impairment by means of odds ratio’s. Furthermore, we examined whether the selection of NCF-tests in the included studies was related to the selection of patients with respect to a specific tumor location. For example, studies focusing on tumors in the left temporal lobe may preferably included tests for language, thereby giving a biased view of the patients’ NCF in this study (i.c. more impairments in the language domain). To detect such bias, we assessed the relationship between patient inclusion based on tumor location on the one hand and domains that were tested on the other by means of Fisher exact test.

## Results

### Search results

**Fig. 2:**
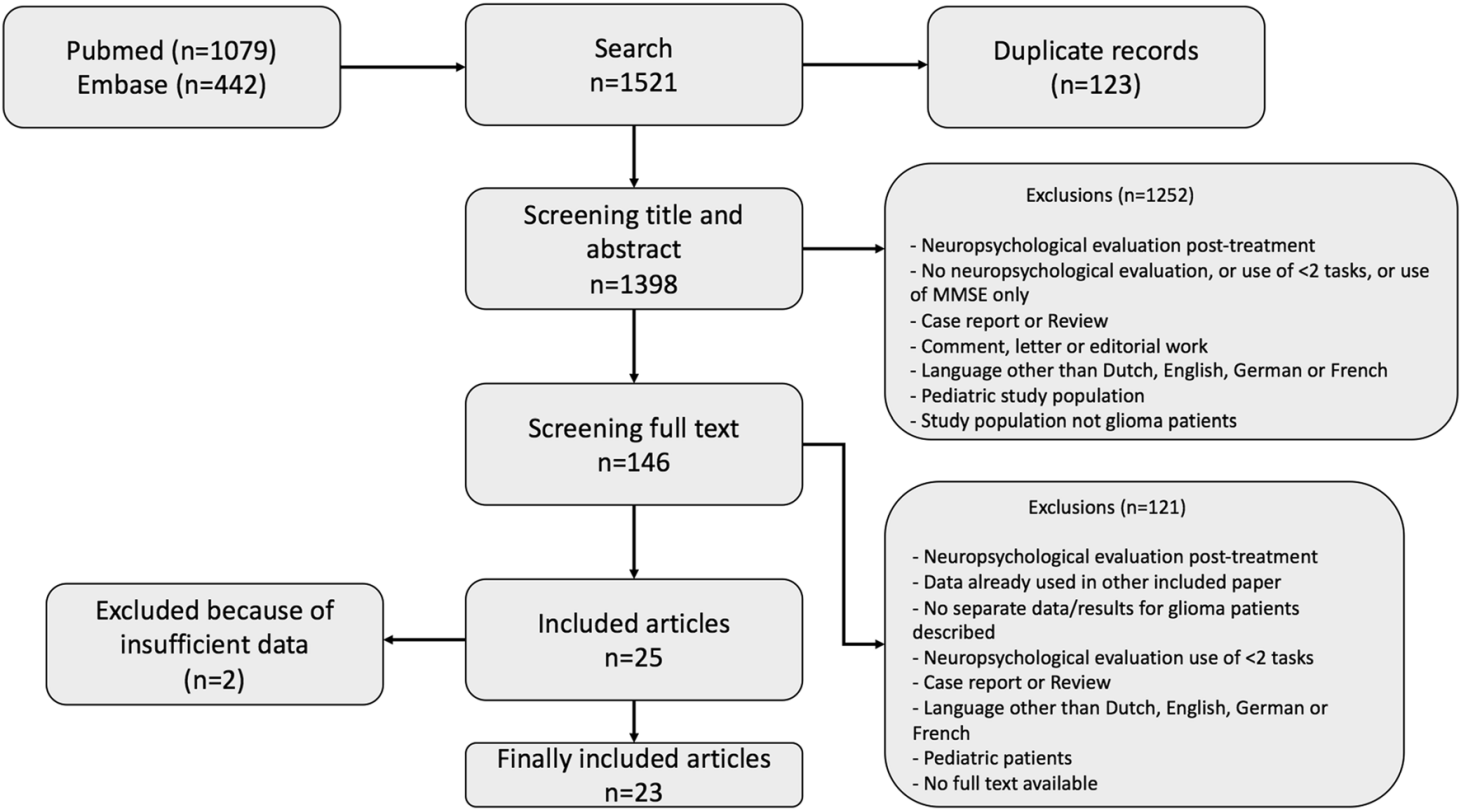
Flowchart of the systematic literature search

The systematic search resulted in 1521 hits, from which 23 studies were included (Fig. 2) [2, 3, 18–38]. The main characteristics of the included studies are summarized in Table 1 (data per study are presented in appendix 2 in supplementary material). Most studies were small observational prospective cohort studies. Patients were selected for tumor location in 11 (47.8%) studies (Table 1).

### Neuropsychological testing

In total, more than 50 different neurocognitive tasks were used to measure NCF in the included studies. Neuropsychological tasks often tap more than one cognitive domain and classification to cognitive domains often differed per study. To allow comparison between studies we made use of predetermined test classification (appendix 3 in supplementary material). Most studies used a combination of neuropsychological tasks (battery), covering more than one cognitive domain. The most frequently tested cognitive domain was executive functioning.

### Neurocognitive functioning (group- and individual-level)

**Fig. 3:**
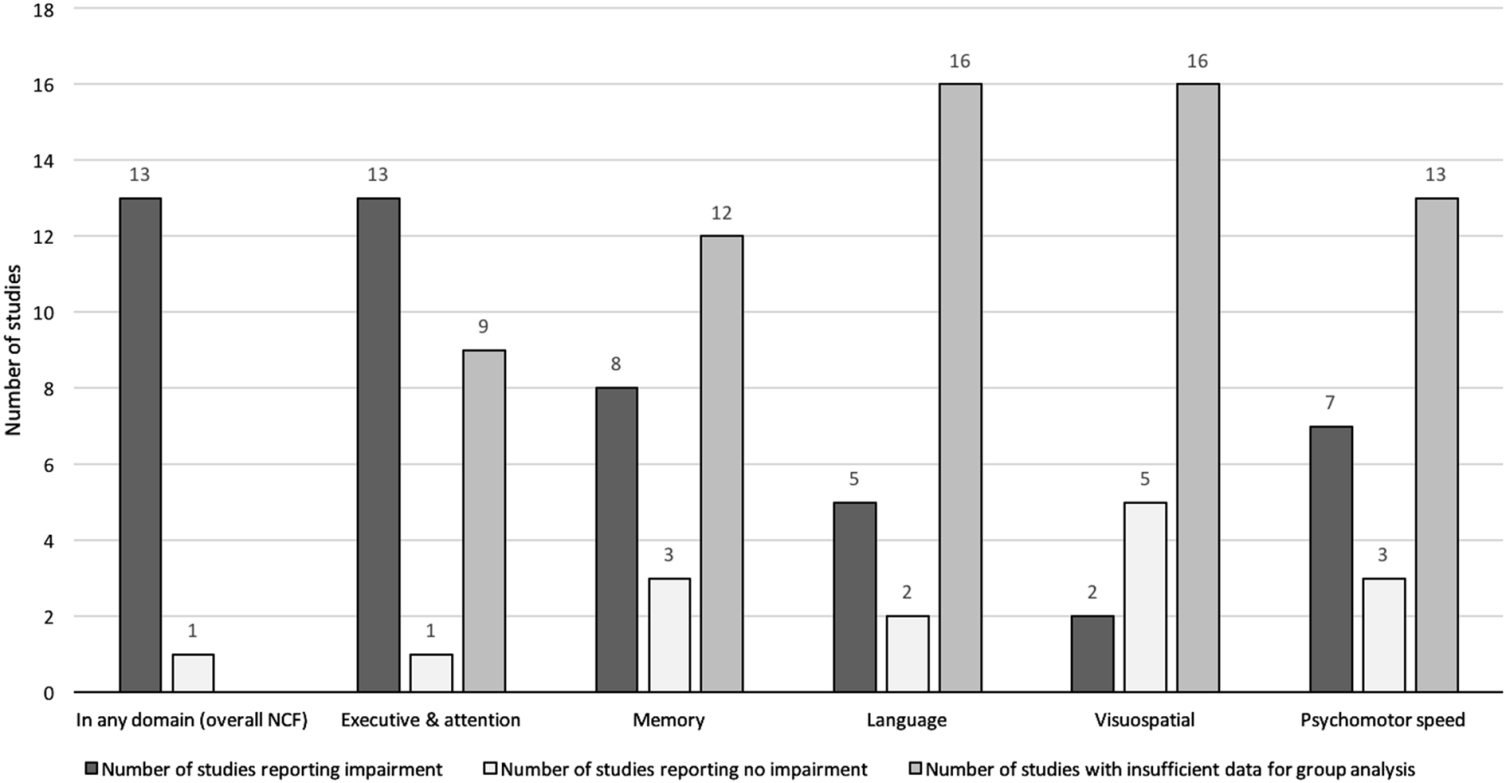
Number of studies reporting—overall and domain-specific—neurocognitive impairment at the group level

**Fig. 4:**
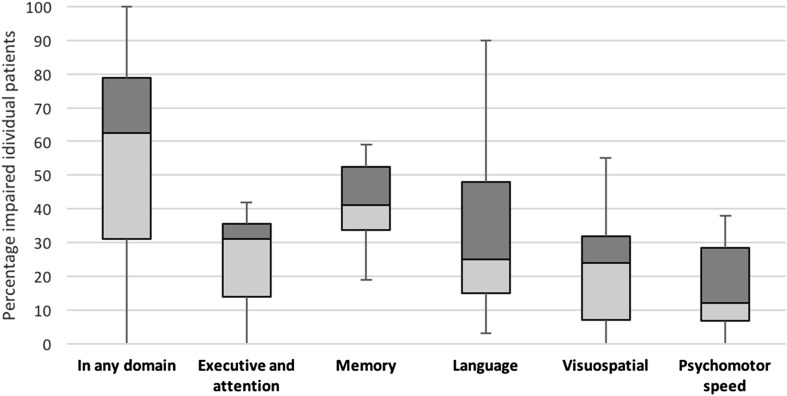
Proportion of glioma patients with cognitive impairment, including overall and domain-specific proportions. The *bars* represent *boxplots* with median, IQR (interquartile range), minimum and maximum values

NCF was analyzed at group level in 14 studies, of which 13 (92.9%) found significantly decreased mean NCF scores in at least one domain (Fig. 3). The proportion of individuals with a cognitive impairment was reported in 15 studies (covering an n = 709 patients, Fig. 4). An impairment in at least one domain, was found in 62.6% of patients (IQR 31.0–79.0) (median percentage across all studies). The proportion of patients impaired in any domain, was independent of the number of domains that was tested (p = 0.07 for 0–2 vs. ≥3 domains tested, independent-sample t-test), but a limited effect cannot be ruled out.

### Domain specific findings (group- and individual-level)

On group-level analysis, 13 studies (of 14) found decreased NCF scores on executive functioning. Only two studies (of seven) found decreased NCF scores on group level for visuospatial functioning. Nevertheless, impairments on individual patient-level were reported for all domains, varying from 12.0% (median speed) to 41.0% (median memory).

### Subgroup analysis

Separate data on NCF for low- versus high-grade glioma were available for seven studies, with a total of 617 patients included. Patients with HGG more often exhibited cognitive impairments compared to patients with LGG (68.9% impaired in HGG versus 31.1% in LGG; OR 2.50; 95% CI 1.71–3.66).

Almost half of the studies (11) included patients based on tumor location and chose specific cognitive domains and tests based on a priori assumptions about functional localization. In studies in which patient selection was based on location, the domain memory was tested less often (OR 0.05; 95% CI 0.01–0.57).

Analyses for other subgroups (for instance based on tumor location, left versus right hemisphere, tumor histology, age, or number of cognitive tasks) were not possible, since sample sizes were too small to permit meaningful analysis.

## Discussion

Cognitive dysfunction in glioma patients has mostly been studied postoperatively. Therefore, the role of the tumor and its direct effects on cognitive functioning is not clear. Through this systematic review of the literature on NCF prior to treatment in diffuse glioma patients, we provide clinicians and researchers in this field a summary of the data on baseline (treatment naive) neurocognitive condition.

We found 23 heterogeneous, mainly small-scale studies with selected groups. NCF was analyzed in 14 studies at the group level, of which 13 studies (92.9%) found significantly decreased mean NCF scores in at least one of the tested domains. The proportion of individuals with a cognitive impairment was reported in 15 studies (covering an n = 709). The median proportion of patients with an impairment in at least one domain was 62.6% (IQR 31.0–79.0). Patients with high-grade glioma more often exhibit cognitive impairments than patients with low-grade glioma (OR 2.50; 95% CI 1.71–3.66).

Based on *group analyses* cognitive impairment appears to occur in the large majority of glioma patients prior to any anti-tumor treatment, and impairments can be found across all main cognitive domains. These findings support the hypothesis that the tumor and the direct effects of this tumor affects NCF in diffuse glioma. Looking more closely at the *level of individual patients*, a median proportion of impaired patients of 62.6% indicates that there is a considerable proportion of patients (one in every three) *without* clear cut cognitive impairments prior to treatment. Speculatively, this variability in the occurrence of cognitive impairment in treatment-naive glioma patients could be explained by tumor-related factors such as tumor location, tumor volume, mass effect of the tumor, WHO grade, histology and molecular markers. Concerning molecular markers, Wefel et al. recently found a complex interrelationship between patients’ NCF, growth velocity and the presence or absence of an IDH-mutation [38]. In addition, Correa et al. found an association between (germline) genetic polymorphisms and NCF in glioma patients, underscoring the role of variability in individual patients’ vulnerability for glioma-related cognitive dysfunction [39]. The available data were insufficient to formally test for the association of these tumor-related factors with NCF across studies.

The problem of neurocognitive dysfunction in glioma is often considered to be most relevant in LGG patients, given their relatively long survival and (often) young age. Analysis for subgroups based on glioma grade showed that HGG patients more often exhibited cognitive impairments than patients with LGG. For interpretation of this finding, it should be taken into account that this analysis was limited to eight studies. No separate data was available for the other included studies. Still, this finding supports the notion that adequate monitoring and treatment of cognitive symptoms is also of great importance in high-grade glioma patients.

This review demonstrates that the available literature is very heterogeneous with regard to the neuropsychological tasks used in the different studies. This limited the possibilities for synthesis of neurocognitive data. Our finding of heterogeneity in neuropsychological tests is supported by a recent systematic review of methods of cognitive assessment in glioma research [40]. To allow comparison across studies, we had to make use of a predetermined classification based on international standard and experience within our group (appendix 3 in supplementary material) to align classifications [17]. Given that each neuropsychological test taps more than one cognitive domain, differences can be found between classification in the original paper and our analyses. Therefore interpretation with respect to what cognitive domain is the most affected needs to be done with caution. However, the finding that impairments can be found in *every* cognitive domain is an important conclusion that is not affected by a possible bias as a result of our predetermined classification.

Another problem we faced was that most studies that reported on the proportion of patients with an impairment for each cognitive test, did not report on the number of *individual* patients that had below-threshold scores for impairment in at least one test within a certain cognitive domain. Therefore, we were not able to determine the exact frequency of patients with impairment within a certain domain. Our method of estimation of this frequency probably resulted in an underestimation of this frequency. Despite the limitations of these methodological choices, the consistent use of our methodology results in systematically obtained estimations of NCF at the group-level and individual patient-level. In determining the frequency of group—and individual-level of cognitive deficits, we decided to consider a domain to be affected on group level, if the mean-score of the group was statistically significant lower compared to norm/control data on any of the tests in that domain and on individual level if the patient performed below threshold on any of the available tests from this domain. By doing this we are able to take all cognitive tasks into account that have been reported, in addition we use rather stringent criteria of -2SD to indicate impairments. Although this approach carries the risk of overestimation of the severity of abnormalities, we feel that it is the best method to value test performance. Each individual test represents a specific function within a cognitive domain; therefore, we considered that one abnormal test—even with other tests results within normal-range—already represents dysfunction in that give domain. For all other methodological choices, we chose options that lead to possible underestimation of the actual cognitive deficits rather than overestimation. Altogether, our summary measures can be viewed as a conservative estimation of neurocognitive abnormalities in treatment-naïve patients with a diffuse glioma.

Furthermore, the risk of bias in the selected studies is high, because these studies were small observational studies that mostly selected patients based on certain glioma locations (selection on location in 11 studies, 47.8%). This selecting on location can lead to selection bias. For example, patients with a tumor located in the left temporal lobe have an increased chance of disturbances in the domain of language, leading to an overestimation of language dysfunction in glioma patients. Further, selection based on location led to more focus on specific cognitive domains such as language and visuospatial functioning and to a lesser extent on more widely distributed functions, such as executive functioning and memory. Unfortunately, the small sample sizes prohibited subgroup analysis of NCF for different glioma locations.

Another limitation is that another form of selection bias may have played a role; In five studies neuropsychological evaluation is done in patients who are undergoing awake surgery [2, 18, 20, 32, 37]. These are often patients with language dysfunction or a tumor in another eloquent area; hence, such patients are probably over represented in the available studies. Moreover, patients who are affected too severely will not undergo awake surgery, and restrain from comprehensive neuropsychological testing. So, preferably, future studies will include all patients with a diffuse glioma prospectively. If this is not possible, it should at least be described how the sample relates to the overall glioma population.

Finally, another possible confounding factor is the effect of medication. Several drugs may disrupt NCF in glioma patients, especially anti-epileptic drugs. At diagnosis of a glioma, drugs aimed at symptom relief will usually be administered at the shortest possible notice. It is therefore unlikely that someone will perform a study of NCF in patients with a glioma prior to first administration of such medications.

Overall we can conclude that most of the above mentioned limitations lead to an underestimation of cognitive functioning on the individual patient-level and group-level per domain and therefore underlines the importance of acknowledgment of the pre-treatment level of NCF. Only the selection of patients by tumor location may lead to an overestimation of cognitive problems in specific domains (for example language).

### Further research and clinical recommendations

Standardization of neuropsychological testing in research on NCF would increase the strength of this research, because outcome measures would be more comparable, permitting formal meta-analysis of neurocognitive data. Recent studies from the EORTC, NRG and other major research institutions have adopted a concise testing battery on several key domains for repeated evaluation of NCF in clinical studies of brain tumors. Further research on (pre-treatment) NCF in glioma patients should contain larger study samples with glioma patients that are not selected based on tumor location, type of surgery or tumor grade. Besides this, etiological research into tumor- and patient-related factors that cause neurocognitive dysfunction in these patients are needed.

For clinical purposes the role of a standardized neuropsychological assessment for the detection of cognitive deficits is well established. Our results underscore the need to pay specific attention to the domain of executive functioning. Detection of executive and other cognitive deficits can lead to optimal counselling of patients and their families, and initiation of specific therapy or training [41]. In addition, neuropsychological assessment may help to monitor cognitive function during awake surgery. However, as previously outlined, it is still unclear which patient-, tumor-, and treatment-related factors play a role in the clinical course of these features; such knowledge is needed in order to develop clinical strategies to optimize cognitive outcome in glioma patients.

## Conclusion

The literature about NCF in diffuse glioma patients prior to anti-tumor treatment is heterogeneous with regard to patient characteristics (glioma location and grade) and neuropsychological tests used in the different studies. Risk of bias is high, because of the small sample sizes and certain patient selection criteria. Based on the *individual patient data*, cognitive impairment occurs in more than half of all glioma patients prior to any anti-tumor treatment, across all main cognitive domains. Based on *group analyses* cognitive impairment appears to occur in the large majority of glioma patients prior to any anti-tumor treatment. These findings support the hypothesis that the tumor itself contributes significantly to NCF dysfunction in diffuse glioma. The variability in the occurrence of cognitive impairment in treatment-naive glioma patients remains largely unexplained. Research on the influence of tumor-related factors (e.g. localization, volume, grade, histology and molecular profile) is necessary in order to explain this variability. Deeper knowledge of the degree and origins of tumor-related cognitive dysfunction will likely facilitate the development of new strategies for treatment and rehabilitation. Furthermore, it is important to take the existence and extent of pre-treatment cognitive dysfunction into account in the research focusing on cognitive sequelae of treatments. In current clinical practice, adequate detection of cognitive dysfunction, especially in executive functioning, may guide the counseling and symptomatic treatment of patients.

## Electronic supplementary material

Below is the link to the electronic supplementary material.

Supplementary material 1 (DOC 83 KB)

Supplementary material 2 (DOCX 21 KB)

Supplementary material 3 (DOCX 95 KB)

Supplementary material 4 (DOCX 16 KB)
