# Supplementary material for: Tumor-related neurocognitive dysfunction in patients with diffuse glioma: a systematic review of neurocognitive functioning prior to anti-tumor treatment
Source: J Neurooncol. 2017 May 31;134(1):9–18. doi: 10.1007/s11060-017-2503-z (PMC5543199; doi:10.1007/s11060-017-2503-z)
Supplement: Supplementary file 2 — Supplementary material 2 (DOCX 21 KB) [file 11060_2017_2503_MOESM2_ESM.docx]

**Appendix 2** Main characteristics per included article

| Study | Study type | Sample size | Aim | Patient population | Cognitive domains | Definition of impairment given? |
| --- | --- | --- | --- | --- | --- | --- |
| Wefel et. al. (2016) | Retrospective cohort study | 119 | Investigate whether IDH status is correlated with neurocognitive functioning | HGG | EA, M, L, S, V | Yes; -1,5SD |
| Cochereau et. al. (2016) | Retrospective cohort study | 15 | Investigate whether neurocognitive impairment exists in a homogeneous LGG population | LGG | EA, M | Yes; -1,65SD |
| Liu et. al. (2015) | Retrospective cohort study | 233 | Investigate whether genetic variation is associated with neurocognitive function in glioma patients | LGG+HGG | EA, M, S | Yes; -1,5SD |
| Noll et. al. (2014) | Retrospective cohort study | 103 | Examine relationships between mood disturbance and neurocognitive functioning with quality of life in glioma patients prior to treatment | temporal lobe glioma | EA, M, L, V, S | Yes; -1,5SD |
| Racine et al. (2014) | Retrospective cohort study | 22 | Examine presurgical baseline cognitive deficits in a case series of LGG patients and determine cognitive effects of surgical resection | LGG | EA, M, L, S, V | No; we used available data for a threshold of -2SD |
| Charras et al. (2014) | Prospective cohort study | 20 | Investigating impact of tumor presence and tumor resection on the functional organization of the brain networks for attention with fMRI | LGG in right hemisphere | EA, V | No; we considered patients impaired if they were classified as impaired in article |
| Habets et al. (2014) | Prospective cohort study | 62 | Investigating tumor and surgical effects on NCF in patients with HGG. | HGG | EA, M, S, V | Yes; -1,5SD |
| Bryszewski et al. (2013) | Prospective cohort study | 20 | Analyzing rearrangement in motor and sensory cortex with fMRI, and its relation with neuro(psycho-)logical status | LGG in motor or sensory cortex | EA, S | Yes; -1SD |
| Satoer et al. (2014) | Prospective cohort study | 45 | Investigate the long-term effect of surgery and potential tumor-related risk factors on different cognitive domains | glioma | EA, M, L, S | - |
| Hu et al. (2013) | Cross-sectional study | 20 | Analyzing ‘small world’ characteristics of resting-state brain networks with MEG in relation to cognitive dysfunction | glioma | EA, M | - |
| Dellen et al. (2012) | Prospective cohort study | 25 | Relating MEG characteristics of brain networks to seizure frequency and cognitive performance | glioma | EA, M | - |
| Mu et al. (2012) | Case-control study | 11 | Exploring the presence of working memory deficits and the identification of facial expressions | glioma in the left frontal lobe | EA, V, S | - |
| Satoer et al. (2012) | Prospective cohort study | 28 | Investigating whether glioma surgery in eloquent areas influences cognition early after surgery | glioma in the left hemisphere | EA, M, L, V, S | - |
| Santini et al. (2012) | Prospective cohort study | 22 | Determining the changes in non-language cognitive domains after awake tumor surgery. | glioma in the left hemisphere | EA, M, L, S | No; we considered patients impaired if they were classified as impaired in article |
| Bizzi et al. (2012) | Prospective cohort study | 19 | Investigating the correlation between language deficits and glioma location | HGG in the left frontal lobe | EA, L | No; we considered patients impaired if they were classified as impaired/aphasia in article |
| Miotto et al. (2011) | Prospective cohort study | 27 | Investigating the NCF in patients with LGG and HGG before surgical intervention | glioma | EA, L, V | - |
| Talacchi et al. (2011) | Prospective cohort study | 29 | Establishing the effects of tumors and their surgical treatment on NCF. | glioma | EA, M, L, S | Yes; considered impaired if the test score was below a cut-off determined on the basis of results obtained in a sample of normal subjects |
| Ruge et al. (2011) | Prospective cohort study | 33 | Evaluating HRQOL and NCF in patients with LGG prior to therapy | LGG | EA, M | - |
| Ek et al. (2010) | Prospective cohort study | 16 | Identifying and characterizing neurocognitive dysfunction at early stages of disease | LGG | EA, M, V, S | Yes; Deficit Scores were derived from the *z*-scores and were considered in the impaired range when lower than - 1 *z*-score |
| Campanella et al. (2009) | Prospective cohort study | 20 | Gain insight in the mechanisms and the anatomical underpinnings of semantic access disorders | glioma in the temporal lobe | EA, V | Yes; below normal range compared to controls (as used in article) |
| Le Rhun et al. (2009) | Prospective cohort study | 15 | Determining pre-treatment NCF in glioma patients | grade II or III glioma | EA, M, S | No; we considered patients impaired if they were classified as impaired in article |
| Teixidor et al. (2007) | Prospective cohort study | 23 | Evaluating the effect of glioma and resection on cognition | LGG in language areas  ­­­­­­­­­­ | L | Yes; -2SD |
| Thomson et al. (1997) | Prospective cohort study | 13 | Determining whether stereotactic biopsy causes a deterioration of language functions | glioma in the dominant hemisphere | L | No; we used available data for a threshold of -2SD |
