# Supplementary material for: Tumor-related neurocognitive dysfunction in patients with diffuse glioma: a systematic review of neurocognitive functioning prior to anti-tumor treatment
Source: J Neurooncol. 2017 May 31;134(1):9–18. doi: 10.1007/s11060-017-2503-z (PMC5543199; doi:10.1007/s11060-017-2503-z)
Supplement: Supplementary file 3 — Supplementary material 3 (DOCX 95 KB) [file 11060_2017_2503_MOESM3_ESM.docx]

**Appendix 3** Predetermined system of allocation of neuropsychological tests to domains in our review.

* These neuropsychological tasks were used in three studies or more.

| Executive & Attention | Memory | Language | Visuospatial | Psychomotor speed |
| --- | --- | --- | --- | --- |
| Concept Shifting Test  MAE (Multilingual Aphasia examination) - Controlled Oral Word Association  Modified Card Sorting Test  Stroop Task*  Test de Brown Peterson  Testbatterie zur Aufmerksamkeitsprüfung  Sous-test du test d’évaluation de l´attention  Trail Making Test Part B*  Verbal fluency (FAS)*  WAIS (Wechsler Adult Intelligent Scale)-R/III - Digit Span*  WAIS-R/III – Similarities*  WCST (Wisconsin Card Sorting Test) Errors | BVMT (Brief Visuospatial Memory Test)-R Totall Recall  BVMT-R Delayed Recall  British Object Recognition Battery  Brief Visual Memory Test  Buchske test verbal memory  Category fluency: animals  CVLT-II (California Verbal Learning Test) long delay recall  CVLT -II total learning score  CVLT-II trial 1 recall  HVLT (Hopkins Verbal Learning Test) -R - Delayed Recall  HVLT-R- Recognition Discrimination*  Münchner Verbaler Gedächtnistest / Munich Verbal Memory Test  RAVLT (Rey Auditory Verbal Learning Test) -15 Words Test*  Rey-Osterrieth Complex Figure (delayed)*  Spatial Supraspan - Learning  Total Recall  Verbal Learning Test*  Wechsler Memory Scale | 15 Words Test*  Aachenar Aphasia Test - Reading aloud  Aachenar Aphasia Test – Repetition*  Aachenar Aphasia Test – Token test*  Aachenar Aphasia Test - Writing to dictation*  Batteria per l' analisi dei deficit afasici  Boston Diagnostic Aphasia Examination  MAE (Multilingual Aphasia examination) - Visual Naming Test or Boston Naming Test*  MAE - Token Test*  Picture Naming Test  Western Aphasia Battery | Clock Drawing Test  Rey-Osterrieth Complex Figure (copy)  Visual and Object Space Perception battery - "Letters"*  Visual and Object Space Perception battery - "Cube Analysis"*  WAIS (Wechsler Adult Intelligent Scale) - R/III - Block Design* | DKEFS (Delis Kaplan Executive function Test) design fluency % accuracy  DKEFS number sequencing  DKEFS number-letter sequencing  Grip strength difference  Grooved pegboard - all  Letter Digit Modalities Test  Symbol Digit Modalities Test*  Tapping Test  Trail Making Test Part A*  WAIS-R/III - Digit Symbol* |
