# Supplementary material for: Tumor-related neurocognitive dysfunction in patients with diffuse glioma: a systematic review of neurocognitive functioning prior to anti-tumor treatment
Source: J Neurooncol. 2017 May 31;134(1):9–18. doi: 10.1007/s11060-017-2503-z (PMC5543199; doi:10.1007/s11060-017-2503-z)
Supplement: Supplementary file 4 — Supplementary material 4 (DOCX 16 KB) [file 11060_2017_2503_MOESM4_ESM.docx]

| **Methodologic issue** | **Solution** |
| --- | --- |
| 1. Definition of neurocognitive impairment | A test score of -2 SD or worse, compared to normative data  If data for a threshold of -2 SD were not available, we used the classification of impairment that the authors provided |
| 1. Convert the results of the tests into the level of domains | We made use of a predetermined test classification according to international standards |
| 1. Group level analysis: articles did not conclude whether the study sample was significantly affected compared to controls or normative data | We used one sample T-tests or independent sample T-tests respectively, to statistically test patient performances against norm/control performance per neuropsychological test of the provided data in the study |
| 1. Group level: converting results of the different tests of the group into the level of single domains of the group | The respective domain was considered significantly affected, if there was a significantly lower score (as defined under item 3) in *any* one of the tests |
| 1. Individual level: determine and project the percentage of impaired individual patients at the domain level | Within all tests performed, per domain |
| 1. Individual level: articles did not state explicitly whether the given number of impaired patients on specific tests within a domain represented *different individual* patients | We used the results of the test in which most patients were impaired (representing the minimum proportion of patients with impairment for the given domain) See also fig 1. |
| 1. Individual level: summarizing the domain results of the different studies | We calculated a median with interquartile range (IQR) for each domain |

**Box 1: Important methodologic issues**
